# A blinded, controlled trial of objective measurement in Parkinson’s disease

**DOI:** 10.1038/s41531-020-00136-9

**Published:** 2020-11-20

**Authors:** Holly Woodrow, Malcolm K. Horne, Chathurini V. Fernando, Katya E. Kotschet, Treat to Target Study Group, Arup Bhattacharya, Richard Blaze, Andrew Charmley, Belinda Cruse, Stephen Duma, Andrew Evans, Mina Ghaly, Blake Giarola, Amy J. Halliday, Thomas Kimber, Anand Kumar, Alexander Lehn, Kate Lilley, Andrew Ma, Neil Mahant, Salar McModie, Manju Perera, Huiliang Melissa Tang, John W. Tillett, Stephen Tisch, Antony Winkel, Christine Wools

**Affiliations:** 1 Florey Institute of Neuroscience and Mental Health Parkville, Melbourne, VIC 3010 Australia; 2 University of Melbourne Parkville, Melbourne, VIC 3010 Australia; 3 St Vincent’s Hospital Melbourne Fitzroy, Melbourne, VIC 3065 Australia; 4 Goulburn Valley Health, Shepparton, VIC Australia; 5 Monash University, Melbourne, VIC Australia; 6 Alfred Health, Melbourne, VIC Australia; 7 Princess Alexandra Hospital, Woolloongabba, QLD Australia; 8 Royal Melbourne Hospital, Parkville, VIC Australia; 9 Westmead Hospital, Sydney, NSW Australia; 10 University of Sydney, Sydney, NSW Australia; 11 St Vincent’s Hospital, Sydney, NSW Australia; 12 University of Notre Dame, Sydney, NSW Australia; 13 Beach Brain, Birtinya, QLD Australia; 14 The Royal Adelaide Hospital, Adelaide, SA Australia; 15 Royal Hobart Hospital, Hobart, TAS Australia; 16 University of Tasmania, Hobart, TAS Australia

**Keywords:** Parkinson's disease, Outcomes research

## Abstract

Medical conditions with effective therapies are usually managed with objective measurement and therapeutic targets. Parkinson’s disease has effective therapies, but continuous objective measurement has only recently become available. This blinded, controlled study examined whether management of Parkinson’s disease was improved when clinical assessment and therapeutic decisions were aided by objective measurement. The primary endpoint was improvement in the Movement Disorder Society-United Parkinson’s Disease Rating Scale’s (MDS-UPDRS) Total Score. In one arm, objective measurement assisted doctors to alter therapy over successive visits until objective measurement scores were in target. Patients in the other arm were conventionally assessed and therapies were changed until judged optimal. There were 75 subjects in the objective measurement arm and 79 in the arm with conventional assessment and treatment. There were statistically significant improvements in the moderate clinically meaningful range in the MDS-UPDRS Total, III, IV scales in the arm using objective measurement, but not in the conventionally treated arm. These findings show that global motor and non-motor disability is improved when management of Parkinson’s disease is assisted by objective measurement.

## Introduction

Although objective measurements are central to therapeutic decision making in most areas of medicine^1^, this is not the case in Parkinson’s disease (PD), where until recently, continuous objective measurement was lacking. Consequently, therapeutic decisions in PD depend on clinical expertise and the person with PD’s (PwP) ability to report their symptoms. Bradykinesia is the main target of current PD therapy, so objective measurement should ideally measure the severity of bradykinesia, its variation with the timing of therapy, and the presence, timing and severity of dyskinesia. Modern sensors have now provided tools for continuous measurement of bradykinesia and dyskinesia^2^ and in previous pilot studies, we examined the contribution of one of these (the Parkinson’s KinetiGraph or PKG, Global Kinetics Corporation^TM^, Australia) on guiding therapy^3,4^. These studies suggested that PwP gain benefit when PD management is assisted with objective measurement and provided power and inclusion criteria for a controlled study. The study reported here compares the management of PD by doctors using information provided by objective ambulatory measurement and conventional assessment, with management using conventional assessment alone. The primary outcome was changes in the Unified Parkinson’s Disease Rating Scale (MDS-UPDRS) Total score and secondary outcomes were changes in the MDS-UPDRS III subscore, the Parkinson’s Disease Questionnaire 39 (PDQ39) score and the Severity of predominantly Non-dopaminergic Symptoms in Parkinson’s Disease (SENS PD) scale. Successful outcome would be important not only for the usual care of PwP but also an important step towards telemedicine and tele-monitoring of PwP.

## Results

**Fig. 1:**
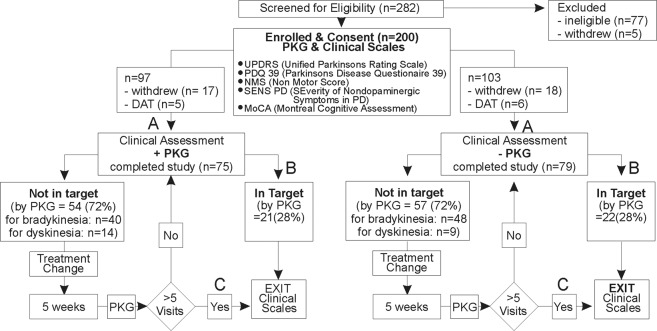
Flow chart showing study design. The first clinical visit and assessment (A) occurs leads to a decision of either “not in target”, treatment change and further clinical assessments or “in target” (B) and clinical scales that constitute the last or final visit. If the subject is still not in target in 5 weeks (C), then the subject exits the study (final visit).

Two hundred and eighty-two participants registered an interest in the study and 200 of these met eligibility criteria and were enrolled in the study (see Fig. 1 and “Methods” for full details of study structure including inclusion and exclusion criteria).

### Comparison of the PKG+ and PKG− arms at entry

There were 97 subjects in the PKG+ arm (assessment of PD made using clinical evaluation as well as with access to PKG information) and 103 in the PKG− arm (assessment of PD made using only clinical evaluation) and 46 were withdrawn during the study: 7 for protocol violations, 11 referred for device-assisted therapy and the remainder were participant-initiated withdrawal for social, personal or non-PD medical reasons. All 46 withdrawn cases were enrolled and had clinical scales and a PKG (which was judged as “out of target” in all cases) prior to the first visit. Nine of the participant-initiated withdrawals occurred before the first visit: the remainder withdrew prior to the final visit, so no final clinical scales or PKG were available from these cases. As the study’s analyses depended on measuring changes from first to final visit, all subject initiated withdrawals and cases referred for device-assisted therapy were excluded from the data set because clinical scales from the final visit could not be obtained. The demographics and scores on clinical scales of PwP who completed the study were similar in the two arms (Table 1). The demographics of enrolled subjects is shown in Supplementary Table 1: there was no difference between enrolled and analysed score of any parameter (statistics not shown) of subjects in each arm. As well, there were no differences in the MDS-UPDRS Total and III scores between subjects attending the clinics or between rural and city subjects (one-way ANOVA, data not shown), with the exception that the scores in rural subjects in the PKG− arm tended to be more severe.

**Table 1:** Demographics of participants who completed study.

	PKG+ *N* = 75	PKG− *N* = 79			
	Mean ± SD	Mean ± SD	*P* value	Δ (SEM)	95% CI
Age	68.2 ± 4.5	67.4 ± 4.9	0.28	0.8 ± 0.8	−0.7 to 2.3
Gender (F/M)	40/35	32/47			
Years to diag.	6.4 ± 4	6.3 ± 3.6	0.90	0.1 ± 0.6	−1.1 to 1.3
LEDD^a^	675 ± 330	760 ± 325	0.11	−85 ± 54	−190 to 20
UPDRS^b^ I	10.5 ± 4.9	11.2 ± 6.1	0.50	−0.6 ± 0.9	−2.4 to 1.2
UPDRS II	9.5 ± 5.8	10.7 ± 6.5	0.27	−1.1 ± 1.0	−3.1 to 0.9
UPDRS III	35.1 ± 9.6	35.8 ± 11.3	0.65	−0.8 ± 1.7	−4.1 to 2.6
UPDRS IV	5.0 ± 3.8	4.6 ± 3.6	0.56	0.4 ± 0.6	−0.8 to 1.5
UPDRS Total	59.6 ± 16.9	62.3 ± 19.6	0.36	−2.7 ± 3.0	−8.6 to 3.1
PDQ^c^ 39	26.9 ± 16.8	29.6 ± 19.1	0.37	−2.7 ± 2.9	−8.4 to 3.1
H&Y^d^	1.9 ± 0.6	2.0 ± 0.6	0.39	−0.1 ± 0.1	−0.3 to 0.1
MoCA^d^	26.5 ± 2.1	25.9 ± 2.5	0.10	0.6 ± 0.4	−0.1 to 1.4
SENS PD^f^	11.1 ± 4.9	12.1 ± 5.2	0.26	−0.9 ± 0.8	−2.6 to 0.7

^a^Levodopa Equivalent Daily Dose, ^b^Movement Disorder Society Unified Parkinson’s Disease Rating Scale. ^c^Parkinson’s Disease Quality of Life 39 Questions. ^d^Hohn and Yahr scale. ^e^Montreal Cognitive Assessment. ^f^Severity of predominantly Non-dopaminergic Symptoms in Parkinson’s Disease.

### Change in clinical scales and PKG from first to last visit in the PKG+ and PGK− arm

**Fig. 2:**
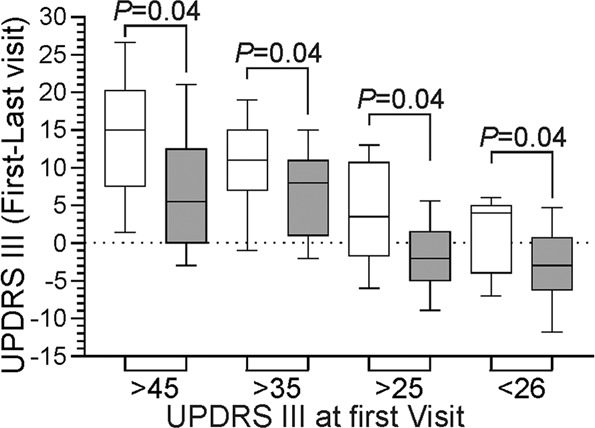
Change in MDS-UPDRS III from first to final visit against MDS-UPDRS III at first visit. The difference between the MDS-UPDRS III at the first and final visit against the severity of MDS-UPDRS III at the first visit (*X*-axis). Plots are box and whiskers (centre line: median, bounds of box:25th and 75th percentile and whisker: 10th and 90th percentile) and white boxes indicating the PKG+ arm and the grey boxes the PGK− arm. *P* values are from Mann–Whitney test.

The difference between clinical scores and PKG values at entry and exit of each arm were examined (Table 2). In the PKG+ arm, the MDS-UPDRS Total scores (the primary outcome) improved by 8.5 points (95% CI 3.4–14, *P* = 0.001). In terms of secondary endpoints, the MDS-UPDRS III improved significantly by 6.4 points (95% CI 3.6–9.2, *P* < 0.001), but neither the PDQ39 (4.7, 95% CI −0.2 to 9.5, *P* = 0.07) nor the SENS PD (1.2, 95% CI 0.1–3.6, *P* = 0.13) reached statistical significance. The change in the means of both MDS-UPDRS scores were of moderate clinical importance^5^ (14% and 18%, respectively) and the improvement in PDQ39 scores was 17%. In contrast, the changes in MDS-UPDRS Total, III, PDQ39 and SENS scores in the PKG− arm were not statistically significant (8%, 7% and 13%, respectively). As might be expected, PwP with the highest MDS-UPDRS III scores at the first visit tended to have the greatest improvement at the final visit (Fig. 2). The average change in scores when the first MDS-UPDRS III > 35 was in the range of large clinically important differences in the PKG+ arm but not in the PKG− arm.

**Table 2:** Outcomes in PKG+ and PG− arms.

	1st visit Mean (SD)	Last visit Mean (SD)			
			*P* value	Δ (SEM)	95% CI
PKG+ (*N* = 75)					
UPDRS^a^ Total	59.6 ± 16.9	51.1 ± 14.7	0.001*	8.5 ± 2.6	3.4–14
UPDRS IV	5.0 ± 3.8	3.5 ± 3.0	0.01*	1.5 ± 0.6	0.4–2.6
UPDRS III	35.1 ± 9.6	28.6 ± 7.9	<0.001*	6.4 ± 1.4	3.6–9.2
UPDRS II	9.5 ± 5.8	9.2 ± 5.5	0.74	0.3 ± 0.9	−1.5 to 2.1
UPDRS I	10.5 ± 4.9	9.9 ± 4.9	0.41	0.7 ± 0.8	−0.9 to 2.2
PDQ39^b^	26.9 ± 16.8	22.2 ± 13.5	0.07	4.7 ± 2.5	−0.2 to 9.5
SENS PD^c^	11.1 ± 4.9	9.9 ± 4.6	0.13	1.2 ± 0.8	−0.3 to 2.7
NMSQ^d^	8.8 ± 5.2	8.4 ± 5.3	0.70	0.3 ± 0.9	−1.3 to 2.0
LEDD^e^	675 ± 330	799 ± 380	0.04*	−124 ± 58	−237 to −9.5
MoCA^f^	26.5 ± 2.1	26.8 ± 2.3	0.42	−0.3 ± 0.4	−1.0 to 0.4
mBKS^g^	25.2 ± 5.5	23.8 ± 3.8	0.08	1.4 ± 0.8	−0.1 to 2.9
AmBKS^h^	22.6 ± 5.3	21.1 ± 3.9	0.046	1.5 ± 0.8	0.0 to 3.0
PTOT^i^	19.9 ± 17.5	13.0 ± 11.0	0.005*	6.9 ± 2.4	2.2 to 12
PTT^j^	4.9 ± 8.8	2.9 ± 5.2	0.10	2.0 ± 1.2	−0.3 to 4.3
PTI^k^	6.2 ± 5.3	5.5 ± 4.9	0.36	0.8 ± 0.8	−0.9 to 2.4
mDKS^l^	3.3 ± 3.7	3.2 ± 2.7	0.79	0.1 ± 0.5	−0.9 to 1.2
PTD^m^	11.5 ± 10.8	12.8 ± 9.5	0.44	−1.3 ± 1.7	−4.6 to 1.9
PKG− (*N* = 79)					
UPDRS Total	62.3 ± 19.8	57.4 ± 17.6	0.10	4.9 ± 3.0	−1.0 to 10.7
UPDRS IV	4.6 ± 3.7	4.0 ± 3.4	0.24	0.7 ± 0.6	−0.4 to 1.8
UPDRS III	35.8 ± 11.4	33.2 ± 10.1	0.13	2.6 ± 1.7	−0.7 to 6.0
UPDRS II	10.7 ± 6.6	9.8 ± 6.2	0.39	0.9 ± 1.0	−1.1 to 2.9
UPDRS I	11.2 ± 6.1	10.6 ± 4.9	0.52	0.6 ± 0.9	−1.2 to 2.3
PDQ39	29.6 ± 19.2	25.6 ± 18.4	0.19	4.0 ± 3.0	−1.9 to 9.9
SENS PD	12.1 ± 5.3	11.4 ± 5.0	0.45	0.60.8	−1.0 to 2.2
NMSQ	9.7 ± 4.9	8.8 ± 4.7	0.26	0.9 ± 0.8	−0.6 to 2.4
LEDD	760 ± 327	870 ± 376	0.06	−110 ± 56	−220 to 0.9
MoCA	25.9 ± 2.5	26.6 ± 2.8	0.14	−0.6 ± 0.4	−1.5 to 0.2
mBKS	26.4 ± 5.8	25.4 ± 5.6	0.24	1.1 ± 0.9	−0.7 to 2.9
AmBKS	23.9 ± 5.6	23.1 ± 5.6	0.35	0.8 ± 0.9	−0.9 to −2.6
PTOT	24.6 ± 20.9	20.3 ± 18.3	0.18	4.2 ± 3.1	−1.9 to 10.4
PTT	5.8 ± 8.1	4.5 ± 6.9	0.31	1.2 ± 1.2	−1.1 to 3.6
PTI	6.5 ± 6.4	6.6 ± 6.1	0.96	0.0 ± 1.0	−2.0 to 1.9
mDKS	3.1 ± 4.5	3.2 ± 3.6	0.87	−0.1 ± 0.7	−1.4 to 1.2
PTD	10.4 ± 12.3	12.7 ± 11.6	0.24	−2.3 ± 1.9	−6.0 to 1.5

*P < 0.05.

^a^Movement Disorder Society Unified Parkinson’s Disease Rating Scale. ^b^Parkinson’s Disease Quality of Life 39 Questions. ^c^Severity of predominantly Non-dopaminergic Symptoms in Parkinson’s Disease. ^d^Non-Motor Symptoms Questionnaire. ^e^Levodopa Equivalent Daily Dose. ^f^Montreal Cognitive Assessment. ^g^Median of 2 min Bradykinesia Scores. ^h^Median of 2 min Active Bradykinesia Scores; ^i^Percent time over target. ^j^Percent time in tremor. ^k^Percent time immobile (day time sleep surrogate): mDKS. ^l^Median of 2 min Dyskinesia Scores: PTD. ^m^Percent time in dyskinesia.

A direct comparison of the two arms was then made (Table 3). The final MDS-UPDRS Total scores in the two arms were statistically different (Table 3, All participants’ Final scores PKG+ vs PKG− (6.3, 95% CI −1.3 to 11.4, *P* = 0.02)) as were the final MDS-UPDRS III (4.6, 95% CI −1.8 to 7.4, *P* = 0.002). SENS PD failed to reach significance (1.5, 95% CI 1.5–3.0, *P* = 0.055) and PDQ39 was not significantly different (3.3, 95% CI 1.8–8.4, *P* = 0.21). The difference between the first and last visit were then calculated for each arm and then compared (Table 3, All participants PKG+ (1st–last visit) vs PKG− (1st–final visit)): the MDS-UPDRS III was significantly different (4.1, 95% CI 1.3–6.9, *P* = 0.01) but the difference for MDS-UPDRS Total, while still large, fell just short of significance (4.4, 95% CI 0.6–8.4, *P* = 0.07).

**Table 3:** Comparison between the PKG+ and PKG− arms of primary and secondary endpoints.

	PKG+ (mean ± SD)	PKG− (mean ± SD)	*P* val	ΔSEM	95% CI
All participants’ final scores. PKG+ vs PKG−					
UPDRS^a^ Total	51.1 ± 14.8	57.4 ± 17.5	0.02*	6.3 ± 2.6	−1.3 to 11.4
UPDRS III	28.6 ± 8.0	33.2 ± 10.0	0.002*	4.6 ± 1.4	−1.8 to 7.4
PDQ39^b^	22.2 ± 13.6	25.6 ± 18.8	0.21	3.3 ± 2.6	1.8–8.4
SENS PD^c^	9.9 ± 4.6	11.4 ± 4.9	0.055	1.5 ± 0.8	1.5–3.0
All participants. PKG+ (1st–last visit) vs PKG− (1st–final visit)					
UPDRS Total	8.1 ± 13.6	3.8 ± 10.2	0.07	4.4 ± 1.9	0.6–8.4
UPDRS III	5.6 ± 8.2	1.5 ± 9.6	0.01*	4.1 ± 1.4	1.3–6.9
PDQ39	5.1 ± 10.6	4.6 ± 9.2	0.85	0.52 ± 1.6	−2.6 to 3.6
SENS PD	1.1 ± 4.2	0.6 ± 9.3	0.49	0.52 ± 1.2	−1.7 to 2.8
Out of target at 1st visit. PKG+ (1st–last visit) vs PKG− (1st– last visit)					
UPDRS Total	10.4 ± 12.7	3.1 ± 9.9	0.0019*	7.3 ± 2.0	3.4–11.2
UPDRS III	7.1 ± 7.0	1.8 ± 9.9	0.0004*	5.3 ± 1.5	2.4–8.3
PDQ39	6.1 ± 9.8	4.0 ± 8.5	0.335	2.1 ± 1.6	−1.1 to 5.2
SENS PD	1.7 ± 4.2	0.8 ± 9.1	0.26	0.92 ± 1.2	−1.5 to 3.3
Bradykinesia at 1st visit. PKG+ (1st– last visit) vs PKG− (1st–last visit)					
UPDRS Total	12.7 ± 12.2	4.8 ± 10.3	0.0027*	7.9 ± 2.2	3.5–12.3
UPDRS III	8.6 ± 8.3	2.6 ± 9.8	0.0008*	6.0 ± 1.8	2.5–9.5
PDQ39	6.8 ± 11.2	2.9 ± 8.8	0.065	3.9 ± 2.0	0.0–7.8
SENS PD	2.2 ± 4.5	0.4 ± 9.6	0.052	1.8 ± 1.5	−1.1 to 4.7

Δ = difference. *P < 0.05.

^a^Movement Disorder Society Unified Parkinson’s Disease Rating Scale. ^b^Parkinson’s Disease Quality of Life 39 Questions. ^c^Severity of predominantly Non-dopaminergic Symptoms in Parkinson’s Disease.

### Decision to treat compared to PKG findings in the PKG+ and PKG-arms

Doctors in the PKG+ arm were directed to treat according to the PKG report, unless there were clear clinical grounds to act otherwise. Thus, the difference in the concordance between the PKG report and the doctors’ actions in the two arms is an indication of the PKG influence on clinical decisions. Furthermore, instances where doctors in the PKG+ arm did not follow the PKG report are cases where doctors decided that the PKG gave an incomplete clinical picture. To examine this, cases were sorted according to whether their PKG was reported as being in or out of target (Fig. 1). In the PKG− arm, 52% of the 23% cases reported as “in target” were treated (almost all for bradykinesia) whereas only 17% of the 24% cases in the PKG+ arm that were “in target” were treated (mostly for dyskinesia not involving the upper limb). The clinical scores of “in target” cases in the two arms were similar and remained unchanged 3 months later (data not shown). Of PwP whose PKGs were reported as “out of target”, 12% were not treated in the PKG+ arm, whereas 30% were not treated in the PKG− arm.

### Outcomes in subjects who were “out of target” at the first visit

The differences from first to final visit in MDS-UPDRS Total, MDS-UPDRS III, PDQ39 and SENS PD scores of subjects who were “out of target” on the first visit in the PKG+ arm were all significant (Table 4) with moderate clinically meaningful differences in the means^5^. None of these scales changed significantly from first to final visit in the PKG− arm. The difference between the primary and secondary endpoints scores at the first and last visit in subjects who were out of Target (Table 3) or who were bradykinetic at first visit (Table 3) were estimated for each individual and these differences in the PKG+ arm were compared with those in the PKG− arm (Table 3). In those who were out of target, there was a significant difference between the PKG+ and PKG− arm for MDS-UPDRS Total (7.3, 95% CI 3.4–11.2, *P* = 0.002) and III (5.3, 95% CI 2.4–8.3, *P* = 0.0004), but not for PDQ39 and SENS PD (although these approached significance in people who were bradykinetic at onset: *P* = 0.07 and *P* = 0.05 respectively).

**Table 4:** Outcomes in PwP participants whose PKG scores were out of target at first visit.

	1^st^ visit	Last visit			
	Mean (SD)	Mean (SD)	*P* val	Δ SEM	95% CI
PKG + ARM (*N* = 54)					
UPDRS^a^ Total	62.8 ± 16.4	51.1 ± 15.5	<0.001*	11.6 ± 3.0	5.8–17.5
UPDRS IV	5.6 ± 3.8	3.3 ± 3.0	<0.001*	2.3 ± 0.6	1.1–3.6
UPDRS III	36.5 ± 9.4	28.6 ± 8.3	<0.001*	7.9 ± 1.7	4.6–11.2
UPDRS II	10.3 ± 5.9	9.5 ± 6.0	0.48	0.8 ± 1.1	−1.4 to 3.0
UPDRS I	11.0 ± 4.6	10.1 ± 5.0	0.31	0.9 ± 0.9	−0.8 to 2.7
PDQ39^b^	28.1 ± 17.4	22.1 ± 13.8	0.045*	6.1 ± 2.9	0.3–11.8
mBKS^c^	26.4 ± 5.6	24.2 ± 3.7	0.016*	2.2 ± 0.9	0.4–3.9
AmBKS^d^	23.6 ± 5.5	21.5 ± 3.8	0.023*	2.1 ± 0.9	0.3–3.8
PTOT^e^	23.8 ± 18.0	14.3 ± 11.5	0.001*	9.5 ± 2.8	4.0–15.1
PTT^f^	5.4 ± 9.9	3.0 ± 5.9	0.116	2.4 ± 1.5	−0.6 to 5.4
PTI^g^	6.8 ± 5.6	5.9 ± 5.3	0.38	0.9 ± 1.0	−1.1 to 2.9
mDKS^h^	3.0 ± 3.8	3.0 ± 2.4	0.98	0.0 ± 0.6	−1.2 to 1.2
PTD^i^	10.5 ± 11.0	12.1 ± 8.7	0.39	−1.6 ± 1.9	−5.2 to 2.0
LEDD^j^	669 ± 299	823 ± 369	0.02*	−155 ± 63	−278 to 32
MoCA^k^	26.3 ± 2.2	26.4 ± 2.3	0.83	−0.1 ± 0.4	−0.9 to 0.7
NMS^l^	8.9 ± 5.1	8.2 ± 5.3	0.49	0.7 ± 1.0	−1.2 to 2.6
SENS PD^m^	11.7 ± 5.1	9.8 ± 4.6	0.17	1.8 ± 0.9	0.1–3.6
PKG-ARM (*N* = 57)					
UPDRS Total	67.4 ± 19.0	61.1 ± 18.4	0.09	6.2 ± 3.6	−0.9 to 13.3
UPDRS IV	5.2 ± 3.6	4.0 ± 3.4	0.09	1.2 ± 0.7	−0.1 to 2.5
UPDRS III	38.5 ± 10.9	34.8 ± 10.4	0.08	3.7 ± 2.0	−0.3 to 7.7
UPDRS II	11.9 ± 6.6	11.1 ± 6.5	0.56	0.7 ± 1.3	−1.7 to 3.2
UPDRS I	11.8 ± 6.2	11.2 ± 4.8	0.57	0.6 ± 1.1	−1.5–2.7
PDQ39	32.6 ± 20.6	29.5 ± 19.6	0.43	3.1 ± 3.9	−4.5 to 10.7
mBKS	27.3 ± 5.9	25.7 ± 5.9	0.18	1.5 ± 1.1	−0.7 to 3.8
AmBKS	24.6 ± 5.6	23.4 ± 5.7	0.28	1.2 ± 1.1	−0.9–3.3
PTOT	27.9 ± 22.2	21.7 ± 19.1	0.13	6.2 ± 4.0	−1.6 to 14.0
PTT	6.7 ± 8.9	4.8 ± 7.0	0.23	1.9 ± 1.5	−1.1 to 4.9
PTI	6.8 ± 6.7	6.9 ± 6.3	0.91	−0.1 ± 1.3	−2.6 to 2.3
mDKS	2.8 ± 4.4	3.2 ± 3.7	0.55	−0.5 ± 0.8	−2.0 to 1.1
PTD	9.4 ± 12.1	12.8 ± 12.4	0.15	−3.5 ± 2.4	−8.1 to 1.1
LEDD	760 ± 357	933 ± 381	0.02	−173 ± 71	−312 to 33
MoCA	25.8 ± 2.5	26.4 ± 2.9	0.28	−0.6 ± 0.5	−1.6 to 0.4
Base NMS	10.2 ± 5.3	9.4 ± 4.9	0.45	0.7 ± 1.0	−1.2 to 2.7
SENS PD	12.7 ± 5.3	12.4 ± 5.2	0.77	0.3 ± 1.0	−1.7 to 2.3

Δ = difference. *P < 0.05.

^a^Movement Disorder Society Unified Parkinson’s Disease Rating Scale. ^b^Parkinson’s Disease Quality of Life 39 Questions. ^c^Median of 2 min Bradykinesia Scores. ^d^Median of 2 min active Bradykinesia Scores. ^e^Percent time over target. ^f^Percent time in tremor. ^g^Percent time immobile (day time sleep surrogate): mDKS. ^h^Median of 2 min Dyskinesia Scores: PTD. ^i^Percent time in dyskinesia. ^j^Levodopa equivalent daily dose. ^k^Montreal Cognitive Assessment. ^l^Non-Motor Symptoms Questionnaire. ^m^Severity of predominantly Non-dopaminergic Symptoms in Parkinson’s Disease.

### Outcomes in subjects who were “out of target” due to bradykinesia at the first visit

**Fig. 3:**
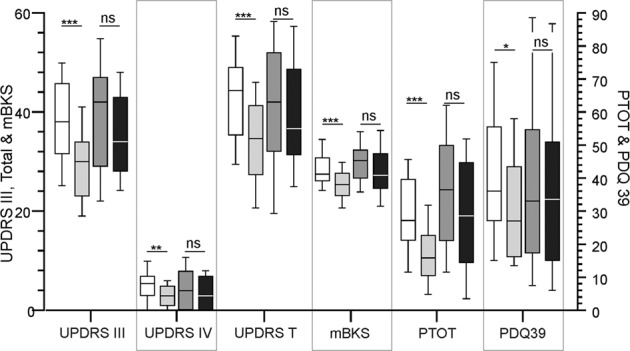
Clinical and PKG scores when “out of target” due to bradykinesia at first visit. These are box and whiskers plots (centre line: median, bounds of box: 25th and 75th percentile and whisker: 10th and 90th percentile) of MDS-UPDRS III, MDS-UPDRS IV, MDS-UPDRS Total, mBKS, Percent time over target (PTOT) and PDQ39 of the first and last visit in the PKG+ arm (white, first visit and light grey, last visit) and PKG− arm (dark grey, first visit and black, last visit) of cases reported as being out of target because of bradykinesia at the first visit. *, ** and *** indicate *P* < 0.05, *P* < 0.01 or *P* < 0.001, respectively, and n.s. indicates not significant (*P* > 0.05). The plot shows a significant reduction in all parameters in the PKG+ scores at last visit.

Subjects in each arm were further segmented into those in whom the PKG report at the first visit (see “Methods, PKG reporting, targets and interpretation”) indicated that the problem was one of either bradykinesia (Category 1, 2, 3, 5: see “Methods”) or dyskinesia (Category 4, 6, 7: see Methods). In cases reported as being out of target because of bradykinesia (Fig. 3), the MDS-UPDRS III, IV and Total and PDQ39 all improved significantly in the PKG+ arm (*n* = 40) but not in the PKG− arm (*n* = 48). The greatest change between first and final MDS-UPDRS III and MDS-UPDRS Total scores in the two arms were in PwP reported as being above bradykinesia target all the time and having dose-related variation (“Out of target” classification 2 on the PKG report). The difference between scores from the first and final visit of each arm were then compared (Table 3, Bradykinesia at 1st visit. PKG+ (1st–last visit) vs PKG− (1st–last visit)). The difference between the mean change in MDS-UPDRS Total in the PKG+ and PKG− arm was 7.9 (95% CI 3.5–12.3, *P* = 0.003, *T*-test) and for MDS-UPDRS III was 6.0 (95% CI 2.5–9.5, *P* = 0.0008, *T*-test). PDQ39 and SENS PD both approached significance (*P* = 0.065 and *P* = 0.052, respectively). There were too few cases where treatment changes were directed at only dyskinesia (*n* = 9 in PKG+ and *n* = 10 in PKG− arms) to examine the effect of using the PKG on management of dyskinesia.

When the PKG scores of all PwP in the PKG+ arm were considered, only the Active mBKS (the PKG score for bradykinesia—see “Methods” for definitions of PKG scores) and percent time over target (PTOT) were significantly changed, but no PKG parameters were significantly different in the PKG− arm (Table 2). When only those PwP whose initial PKG scores were out of target were considered, all the PKGs’ bradykinesia scores (mBKS, AmBKS and PTOT) were reduced significantly in the PKG+ arm but not the PKG− arm (data not shown). In PwP treated for high bradykinesia scores (Fig. 3), there was marked change in the mBKS, AmBKS (not shown) and PTOT. As discussed below, the PKG scores were more sensitive when focussed on changes in bradykinesia.

All subgroup analyses described above were planned prior to the commencement of the study.

### Levodopa equivalent daily dose (LEDD) scores

Although the use of medications was not an endpoint in this study, it is of interest to know whether the differences in outcomes was achieved through differences in medication use. There were large increases in LEDD in both arms (Table 2): PKG+ of 124 LEDD (*P* = 0.04) and in PKG− of 100 LEDD (*P* = 0.06), with no significant difference between the final LEDD in each arm (Δ (SEM) = 61 LEDD, *P* = 0.25, 95% CI = 239, *T*-test). This is relevant because a larger change in MDS-UPDRS Total and III was achieved in the PKG+ arm despite similar final LEDD and a (slightly) larger change (24 mg) in LEDD. Furthermore, the MDS-UPDRS IV fell in both arms, indicating that improvement in bradykinesia could be achieved without an increase in dyskinesia. In the PKG+ arm, D2 agonists were changed in 33% (reduced in 3%) of PwP compared to 18% (reduced in 8%) in the PKG− arm (*P* = 0.0001, Fishers Exact) and with the median change in LEDD being 75 (interquartile range (IQR): 60–150) in the PKG+ arm and 38 (IQR: −163 to 60) in the PKG− arm. The average reduction in levodopa dose interval was ~40 min in the PKG+ arm compared with ~20 min in the PKG− arm (*P* = 0.03, SEM = 18.1 min, 95% CI = 32 min). There was a non-significant trend to increase the number of daily doses in the PKG+ arm. As there were improvements in MDS-UPDRS III and IV in the PKG+ arm (Table 2) using similar LEDD, more targeted use of dopaminergic therapies reflected in changes in the D2 agonist dose and levodopa dose interval are presumably responsible.

### Variability in doctors

**Fig. 4:**
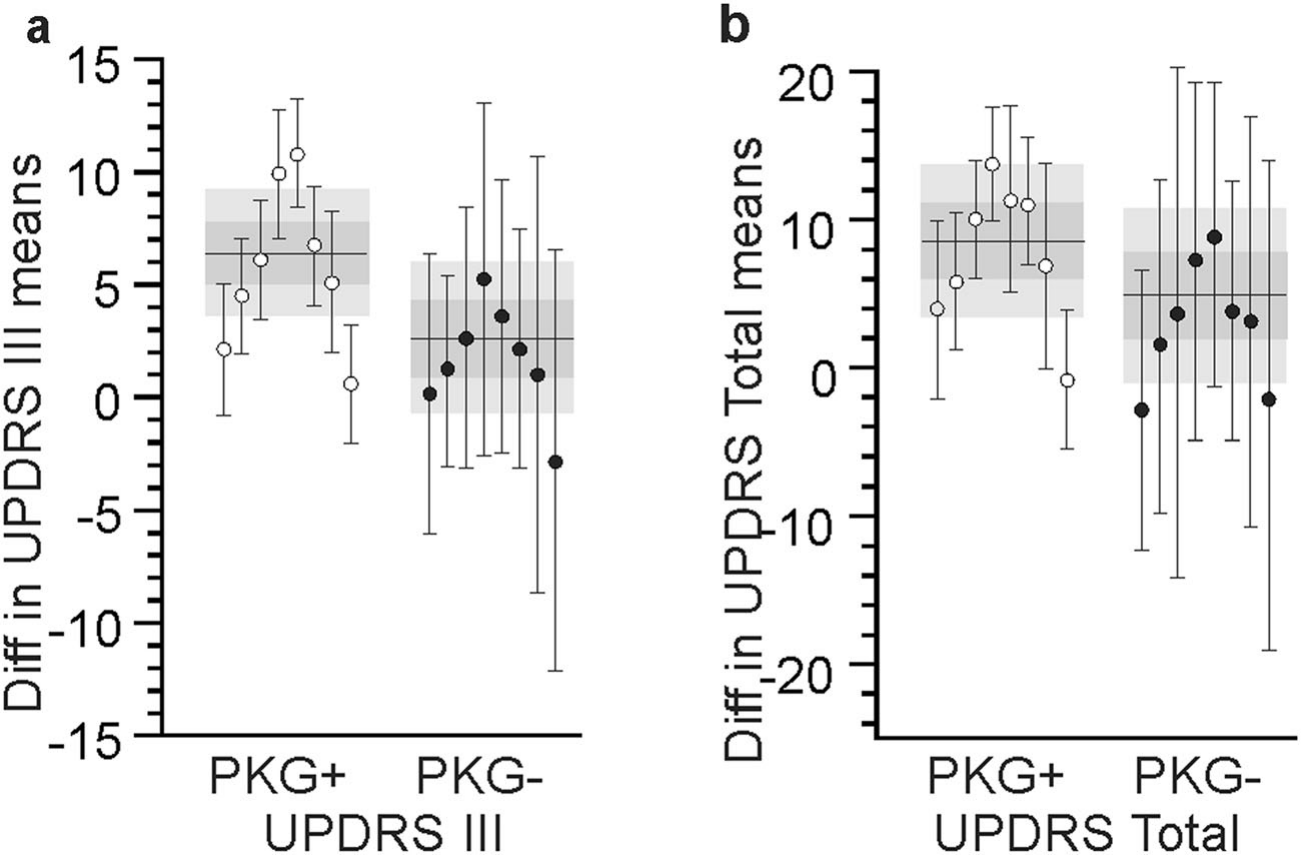
Change in MDS-UPDRS produced by each doctor. Panels a, b show the difference between the means (of the 1st and last visit) of the MDS-UPDRS III (**a**) and MDS-UPDRS total (**b**) for each doctor (each dot is a doctor and the bar is the SEM). White dots represent PKG+ doctors and black dots indicate PKG− doctors. The grey bands indicate the 95% CI of the whole population (PKG+ and PKG− respectively) with the horizontal line indicating the mean and the darker grey the IQR. Note that the 95% CI and SEM are smaller in the PKG+ arms.

Although the effect of the PKG on doctors’ decisions was not an endpoint in this study, it is of interest to know whether individual doctors may have biased the findings. The effect of the PKG on doctors’ decision making was examined by comparing the difference in the mean from the first and last visit of all PwP assessed by an individual doctor with the 95% CI of the whole arm (Fig. 4a, b). Both the confidence intervals of the PKG+ arm and the standard errors of individual doctors in the PKG+ arm were smaller than in the PKG− arm, implying that PKG information resulted in greater consistency in decisions. The distribution of each doctor’s scores lay within the confidence intervals, but the outliers were distributed equally above and below the confidence interval indicating that undue influence on results by one doctor was unlikely. However it does lead to the speculation that the two doctors lying above the confidence interval were greater compliers with the PKG instructions, whereas the two that lay below the confidence interval were least influenced and indeed their scores lay close to the centre of the PKG− 95% CIs. Examination of their documented decisions suggest that this was the case.

Although the median number of visits (2) was the same in both arms, there were more visits in the PKG+ arm (*P* = 0.01, *χ*^2^ test). When only PwP whose PKG was reported as out of target was considered, the median number of visits was one higher in the PKG+ arm (median 3: IQR = 3) compared to the PKG− arm (median = 2 (IQR = 2) and this difference was significant (*P* = 0.0009, *χ*^2^ test).

Subjects referred for device-assisted therapies were excluded from the study because the device-assisted therapies would be unlikely to be implemented and optimised in the time of the study and so any effect of objective therapy would not be captured in the analyses. In general, these subjects’ MDS-UPDRS IV scores (mean 9.2 ± 4.5 SD) and PKG scores (median dyskinesia score (mDKS: mean 8.7 ± 7.6 SD): percent time in dyskinesia (PTD: mean 20.4 ± 13.6 SD, upper limit > 16.2) bradykinesia (PTOT: mean 12.7 ± 9.1 SD, upper limit > 12.4)) were higher than the study population.

## Discussion

The main findings of this study were that therapeutic decisions supported by objective measurement resulted in reduced bradykinesia (MDS-UPDRS III), motor complications (MDS-UPDRS IV) and improved global motor and non-motor disability as measured by the MDS-UPDRS Total (the primary endpoint). The changes in these scores in the PKG+ arm compare favourably with clinical trials of therapy for PD: among pharmacological agents, only levodopa had a similar effect on size of UPDRS III^6,7^ and UPDRS total scores^7,8^ (see Table 5 of ref. ^5^) and 6 months after deep brain stimulation, the UPDRS III in the “on” state (as was done in the current study) improved by 4.0 (±10) MDS-UPDRS points^9^ compared to 6.4 (±1.4) in this study (Table 2). While the change in PDQ39 score in the PKG+ arm just failed to reach statistical significance (*P* = 0.07), the mean change in this study (4.7) compares favourably with that of the best change (~2.2) from levodopa^7^ and the 17% change in PDQ39 in this study also compares favourably with that seen in DBS (24%)^9^. Retrospective power analyses indicate that all the endpoints would have been significant if 100 subjects in each arm had completed the study. However, limited resources and logistics closed the study after 2 years.

The improvements in clinical scores in the PKG+ arm were achieved with increments in LEDD that were similar to those used in the PKG− arm, probably related to greater use of D2 agonists and shorter intervals between doses of levodopa. This suggests that the benefit in the PKG+ arm may have led to more strategic deployment of dopaminergic agents to target complications indicated in the PKG. It is noteworthy that the MDS-UPDRS IV and dyskinesia scores did not deteriorate in either arm with increased dose, which counters the expectation that increasing dopaminergic stimulation will necessarily lead to an increase in dyskinesia.

This study was based on the hypothesis that assessment and treatment using standard methods would not result in a significant improvement, because most PwP in Australia are cared for by a neurologist: indeed 44% PwP in this study were usually treated by a Movement Disorder specialist. Doctors in the PKG− arm did not significantly improve participant’s scores from what their more experienced counterparts in the community had already achieved, most likely because management was already at the best level of “standard of care”. Even so, there were improvements in the MDS-UPDRS III and total scores in the PKG− arm (2.6 and 4.3, respectively, Table 2) that just reached the threshold for minimally clinically important difference (2.5 and 4.3 as threshold for MDS-UPDRS III/Total respectively)^5^. Fellows were chosen for this study to reduce the risk of bias: the similarity in their clinical experience was relatively uniform and more similar than their experienced counterparts, and importantly more likely to comply with the directives of the PKG. Training in neurology in Australia is quite uniform and the experience in PD management of doctors in the two arms was, if anything, slightly greater in the PKG− arm. It is unlikely therefore that a bias due to experience of the doctors would have systematically influenced the outcome. Furthermore, most doctors at the PKG− clinics saw the PKG as an unproven entity and there is no reason to suspect their motivation was other than to treat patients to the best of their capacity.

The inclusion criteria ensured that most subjects would have similar severity of disease and fewer subjects whose motor symptoms could not be treated. Table 1 indicates that there was no statistical difference between the arms and Fig. 2 indicates PwP with the greatest bradykinesia provided the greatest opportunity to gain improvement in scores. All PwP wore a PKG logger prior to each visit and received the usual dose reminders and they were blinded as to whether they attended a PKG+ or PKG− clinic. Furthermore, the balance between PwP from city and regional patients was balanced in the two arms.

The written PKG report was provided to ensure that information recorded by the PKG was available regardless of the PKG+ doctor’s ability to interpret the PKG. Reporters were blinded to which arm the PKG came from or any other knowledge of the PwP and their report was entirely based on information in the PKG. It was designed to support the information being sought from the PwP by clinical assessment, including severity of bradykinesia or dyskinesia, whether there were fluctuations, their timing with respect to dosing and whether the best response to a dose was satisfactory (i.e. in target). These questions are central to any therapeutic intervention. The “out of target” PKG classification was designed to highlight this information and the one-day training programme in treatment of PD which was provided to doctors in both arms discussed in detail the type of clinical response these classifications might require.

Clearly it was not possible to blind the doctors to the information in the PKG; however, PwP were blinded as to whether they were participants in the PKG+ or PKG− arm. Nor, for the reason outlined in the “Methods”, was it possible to have PwP assessed with and without the PKG in one clinic. However, it would seem that PwP were similar in each arm (Table 1), and that the mix of urban and regional participants in each clinic were of similar severity. Because PwP chose to attend the closest clinic and, having chosen to make clinics either PKG+ or PKG− to diminish PKG bias on doctors, the options for randomising subjects to treatment were removed. However, the two arms appear to be similar and the profile of subjects attending each clinic was similar although there was an insignificant trend for greater severity of disease in the PKG− arm, especially rural subjects. Furthermore, increased severity of disease at the first visit implied greater opportunity to make a significant difference providing objective measurement was available (Fig. 3): the difference between the two arms may have been more marked if the PKG+ arm had subjects with greater disease severity at first visit.

The pilot studies that guided the design of this project^4,10^ reported improvement in non-motor scores. This was not found in this study, although the SENS PD scores did approach significance. These pilot studies led to the exclusion of people with low cognitive scores and aged >75 because the risk of non-motor symptoms that contraindicated changing dopaminergic therapy was much higher in this age group. However, it may have also excluded subjects who had therapeutically addressable non-motor symptoms. It is important to note that limits around age and cognitive scores were aimed at reducing the cost and logistics of the study by reducing the number of people who would be enrolled and subsequently be excluded because of referral for device-assisted therapy or contraindications to dopaminergic therapy. We estimate the inclusion criteria to cover approximately 2/3 of the PD population. The exclusion criteria should not be taken to mean that objective measurement is not relevant in excluded subjects: indeed, the results of this study suggest that all subjects in whom changing dopaminergic therapy could be considered warrant measurement. As nearly half the participants were usually managed by movement disorder specialists, it also suggests most PwP would benefit from measurement, regardless of the expertise of their treating clinician.

An individual’s PKG scores relative to the target range drove the decision to treat in the PKG+ arm and the final MDS-UPDRS III score of 28 in this arm reflects treating to this target. Experts can opine on the appropriateness of a UPDRS III score of 28 as a target or whether a lower score would be desirable, but evidence from further studies should set the target. It is relevant that information such as that synthesised within the PKG report relates to the timing and pattern of how and when the BKS exceeds target, relative to medications (Fig. 2). This provides information about dose interval and dose size for levodopa and not simply a binary trigger of whether or not to intervene.

In Table 2, the Active Median Bradykinesia Score (AmBKS) and percent time out of target (PTOT) were the only PKG scores to change significantly in the PKG+ arm. However, in PwP treated for high bradykinesia scores (Fig. 2), changes in mBKS, AmBKS (not shown) and PTOT (Fig. 2) were marked. It is likely that the AmBKS and PTOT, both of which account for inactivity, are more sensitive than other PKG scores, such as mBKS, which do not have this correction. As well, mBKS falls when dyskinesia is present (and vice versa): thus, mBKS increases in individuals treated for dyskinesia, obscuring statistical change in a population where both excess dyskinesia and bradykinesia are being addressed. When only bradykinesia is the target of therapy (Fig. 2), a very clear fall in mBKS was apparent.

In this study, assistance from objective measurement significantly improved therapeutic management of PwP. Considering other areas of medicine, this should not be a surprising finding and suggests objective measurement in routine clinical care and in clinical trials should be considered, including in the era of telemedicine. It raises the question as to whether novel therapies should be compared in populations whose conventional therapy has already been optimised using objective measurement.

## Methods

This blinded, controlled study was approved and overseen by the St Vincent’s Hospital, Melbourne, Human Research & Ethics Committee (approval No. HREC/17/SVHM/298) and conducted between March 2018 and December 2019. Subjects provided written consent according to the Declaration of Helsinki. Study design is described below. This Trial was registered on ANZCTR (https://www.anzctr.org.au/Trial/Registration/TrialReview.aspx?id=373960), registration number ACTRN12618000197235.

### Participating doctors and clinics

The aim of the study was to test whether outcomes for PwP were improved when doctors used information provided by the PKG. Ensuring that doctors were of similar expertise was therefore important, so 15 doctors in advanced neurology training (8 doctors) or recently made Fellows (7 doctors) of the Royal Australasian College of Physicians participated as the doctors in the study. Doctors at this level were chosen because experience in PD management was similar. The numbers having done a Movement Disorder Fellowship in the PKG+ arm was four compared to eight in the PKG− arm whereas the level of training in the PKG+ arm was three still in training and five recently made Fellows compared to four trainees and four Fellows in the PKG− arm. Experience in PD ranged from first year of exposure (trainees) to 2 years for some fellows but overall the experience in PD was higher in the PKG− arm. All doctors attended one day of training in the assessment and management of PD, emphasising the use of history to identify motor and non-motor features of PD, contraindications to and side effects of anti-Parkinson’s medications, and recognition of candidates for device-assisted therapies. Doctors in the PKG+ arm received a further day of training in interpreting the PKG.

All doctors were supervised by a Movement Disorder specialist, thus ensuring that the trainee/fellow worked in a clinic that provided experience in the management of PD, including with device-assisted therapies. In the study, doctors worked independently of the supervisors, whose role was to be available to provide advice on specific management issues. Although participating doctors who were Fellows of the College could practice independently, access to a supervisor is a requirement for trainees. For consistency, therefore all participating doctors had supervisors.

A requirement for enrolment of PwP was that they had not been assessed and managed previously using the PKG. It was also desirable that doctors with experience using the PKG were in the PKG+ arm. The PKG is used almost exclusively in Movement Disorder clinics in Australia, and approximately half of the participating clinics (predominantly in Melbourne) use the PKG extensively and these became the PKG+ clinics. On the other hand, clinics where fellows had infrequent experience with the PKG became PKG− clinics. The study was conducted at 12 clinics (7 in major cities, 3 in regional centres (2 affiliated with a major city service)), equally divided between the PKG+ and PKG− arms. A median of 12 PwP (IQR = 6.5) attended each site, with each doctor seeing an average of 8 PwP. Three doctors worked at two different sites. Note that one doctor was in the PKG− arm in year 1 and the PKG+ arm in year 2.

Most PwP did not usually attend one of the participating clinics and were reluctant to travel across town so they were allocated to the most conveniently located clinic. This meant that randomisation was not possible. However, there were no differences in the MDS-UPDRS Total and III scores between subjects attending the clinics or between rural and city subjects (one-way ANOVA, data not shown), with the exception that the scores in rural subjects in the PKG− arm tended to be more severe. PwP were blinded as to whether they were attending a PKG+ or PKG− clinic and all subjects wore a PKG logger prior to each visit: note that PKGs were performed in each study arm with the only difference being that only the PKG+ doctor had knowledge of the information from the PKG at each visit.

### Study structure

Recruitment was through patient advocacy organisations (state and national Parkinson’s associations and Shake It Up Australia) and social media. Consent to be contacted and preliminary screening was provided on a study specific website. After contact, 282 participants were further screened (Fig. 1) and reviewed for eligibility. Inclusion criteria required PwP to have (a) idiopathic PD of ≥4 years or taking ≥four doses of levodopa/day (because PwP with early disease or fewer doses had a much greater likelihood of being in target^4^); (b) aged 59–75 years (because most PwP under 59 who also met the above criterion were also likely to be candidates for DBS^4^ and PwP aged over 75 had a high incidence of contraindications to increasing dopaminergic therapy^4^); (c) ability to attend a study clinic and willingness to change medication according to the advice of the study doctor. Exclusion included (a) treatment with, or under consideration for, device-assisted therapy; (b) Montreal Cognitive Assessment score ≤ 21 (the aim was not to exclude dementia but to reduce the numbers of PwP whose chances of having contraindications to increasing dopaminergic therapy was high, and previous pilot study^4^ showed that scores ≤21 was the cut off to achieve this); (c) history of orthostatic hypotension or hallucinations or other symptoms that would prevent increases in PD medications; (d) having a previous PKG assessment in routine clinical care. At the screening visit, all consenting and eligible participants were assessed with clinical scales (Fig. 1), medications were recorded, and a PKG logger was provided. PwP were then allocated to the most conveniently located clinic and were blinded as to whether this was a PKG+ arm or PKG− arm: i.e. whether the treating doctor had access to the information from the PKG at each visit. Doctors in neither arm had access to scores from clinical scales, which were performed by the same certified assessor who was blinded to the doctor’s assessments. This was to reduce missing data and assessor variation. MDS-UPDRS III was performed during the screening visit and at the last visit when subjects were in the “ON” state.

At the first consultation, PKG+ doctors assessed PwP using history, examination and PKG information to decide whether the PwP’s motor features were in target (no further treatment required) or out of target (Fig. 1). In the latter case, a plan for changing treatment was provided and a PKG logger was worn prior to the next consultation 5 weeks later. The same assessment protocol was followed until the PKG data were in target: a maximum of five visits were permitted, inclusive of the first assessment. This was designated the “final or last” visit and PwP then exited the study and clinical scales and PKG were performed. PKG− doctors followed a similar protocol, except their assessment was entirely clinical and without access to PKG information. If the PwP refused to change therapy at the first visit they were excluded from the study, as willingness to change medications was part of the inclusion criteria. If there was refusal after several attempts to improve control, then this case was included in the study. Previous pilot studies^3,4^ were used to determine the sample size and selection criteria. These calculations showed that power to achieve the primary endpoint MDS-UPDRS Total would be achieved with 75 in each arm although 100 in each arm was required to achieve power for PDQ39. The study was terminated when there were no more eligible participants volunteering in participating regions.

### The PKG system

The PKG system consists of a wrist-worn data logger, algorithms^11^ that produce data points for bradykinesia (BKS) and dyskinesia (DKS) every 2 min over the 6 days the logger was worn and a series of graphs and scores that synthesise this data into a clinically useful format known as the PKG report. The BKS and DKS are plotted against the time of day and the time when medications are due is also provided. The numerical output is described in detail in other publications^4,10–19^. All outputs are derived from data recorded between 09:00 and 18:00 of the six recording days and those relevant to this study are summarised below. Where relevant, the upper limit obtained from aged matched, non-PD controls is shown in brackets.

mBKS: The median of all BKS from the 6 days while the logger was being worn and PwP was not asleep.

AmBKS (<23): Active mBKS. The median of BKS < 42 (which removes most inactivity^19^ and sleep^20^) from the 6 days.

PTI (>6.5%): Percent time immobile is the proportion of BKS > 80, which correlates with the polysomnographic recordings of sleep^11,19^.

PTOT (>12.4%): Percent time over target^4,21^ is the proportion of time BKS were over target (BKS = 26), not including PTI.

PTT (>1%): Percent time with tremor is the proportion of 2 min epochs containing tremor^22^.

mDKS: The median of all DKS from the 6 days.

PTD (16.2%) Percent time in dyskinesia^4,21^ is the proportion of time that DKS were over target (DKS = 7), not include epochs with high levels of walking.

### PKG reporting, targets and interpretation

**Fig. 5:**
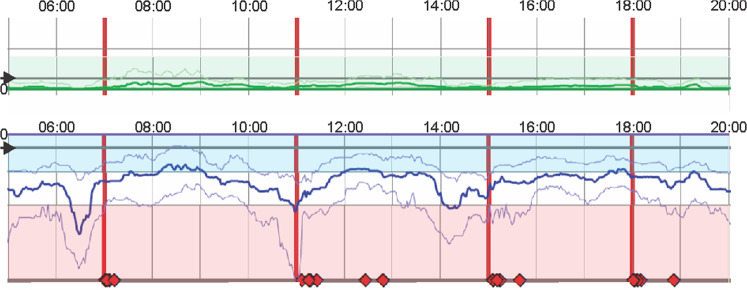
An example of part of a PKG. This shows only the smoothed moving median of the DKS over a 6-day period (darker green line) and BKS (darker blue line) plotted against time of day (*X-*axis). The *Y-*axis shows the severity of DKS (increasing severity upward from the dark green line adjacent to the zero) and BKS (increasing severity downward from the dark blue line adjacent to the zero). The median BKS and DKS of non-PD subjects is shown with the arrow. The shaded green and blue zones show the target range. BKS in the shaded pink region are usually associated with inactivity. The vertical red lines are the times that reminders were delivered, and the diamonds show the time when consumption of medications was acknowledged. In this example the median BKS is outside of target at the time of the first dose. The response to each dose just reaches target and there is “wearing-OFF” after 2 h (1st dose) and ~3 h (2nd dose). This case was reported as having bradykinesia above target with “Bradykinesia only as Wearing Off” according to the 7-point classification described in the text.

All PKGs were reported by one of the authors (K.E.K., M.K.H.) in a standard format, blinded to the study arm. The reporting was qualitative but referred to target ranges that separate “controlled” PD from “uncontrolled” PD, established by a consensus of a panel of four neurologists experienced in treating PD, then trialled in a previous study^4^ and subsequently supported by expert panels^23,24^. The Bradykinesia target was BKS < 26, corresponding to the shaded area in Fig. 5: note that this is different to the mBKS which is the average of this activity of 09:00–18:00, but refers the moving average bradykinesia score over the day being in excess of the target at the point in time. A BKS of 26 corresponds to a UPDRS III of approximately 30 (ref. ^17^). The Dyskinesia target was a DKS < 7 corresponding to an Abnormal Involuntary Movement Score of ~ 9 (ref. ^11^). The study protocol did not require tremor to be treated if PKG bradykinesia scores were in target. The PKG report along with the PKG was available to the PKG+ doctors at the time of each study visit. PKGs were reported as being (a) in target; (b) out of target; (c) likely *out of target* but resolve potential artefact; (d) likely in target but resolve *potential artefact*. If the PKG was reported as “out of target”, a further seven point classification was provided, for the purpose of statistical sub-analyses (see Fig. 5 for reference).

1. Global Bradykinesia. BKS > 26 at all times between 09:00 and 18:00, without dose-related variation: mBKS > 26.
2. Global bradykinesia and wearing off. BKS > 26 at all times between 09:00 and 18:00, but with dose-related variation. mBKS may be >26.
3. Bradykinesia only as wearing off. The BKS > 26 at the time of one or more, for at least 30 min. Re-emergence of tremor is supporting evidence.
4. Peak dose dyskinesia alone. DKS > 7 at the time of one or more doses for at least 30 min, providing not artifactually elevated by walking or exercise.
5. Predominantly bradykinesia, but with peak dose dyskinesia.
6. Predominantly peak dyskinesia dose, but with bradykinesia.
7. Global dyskinesia. DKS > 7 at all times between 09:00 and 18:00, without dose-related variation. mDKS may be >7.

Protocol required doctors in the PKG+ arm to follow the PKG findings when deciding whether to change treatment, according to these criteria unless:

- The doctor’s clinical findings show that the PKG report is incorrect: for example, cervical dyskinesia which would not be detected by the PKG, was observed and treated by the doctor.
- The doctor considered that a referral for device-assisted therapies was indicated because of the severity of motor complications and/or futility of further changes to oral medications.
- A contraindication has been identified, including a reasonable concern that it will be induced by a change in therapy.
- PwP declines further change. As consent was given to change dosage according to protocol, the doctor was required to attempt reasonable persuasion to agree to changes.
- Futility. Attempts at earlier visits to improve scores have failed.
- All five visits have been used.

In the PKG− arm, doctors were required to use standard clinical practice to assess whether treatment was adequate or whether further treatment was required, with the same caveats to changing therapy that applied to the PKG+ arm. In both arms, a review appointment was only for addressing treatable motor symptoms/scores: non-motor symptoms could be treated, but a further “in-study” visit should not be scheduled if the sole aim was to address a non-motor symptom.

### Statistical analysis

Each variable was assessed with a two-tailed, heteroskedastic *t*-test. An exact probability value (*P*) is provided and significance was set at *P* < 0.05. The difference in the means is provided along with a 95% confidence interval (95% CI) for the difference of the means. Statistics are usually quoted as (difference in standard error of means, lower and upper 95%, *P* value). There was no missing data for the primary and secondary outcomes, with the exception of PDQ39, where two data points were missing from the PKG+ arm and three data points from the PKG− arm and was handled by listwise deletion. It was planned prior to the study that the endpoints would be considered for (a) all subjects (in target and out of target) that completed the study (i.e. had first and final visit scores); (b) subjects whose scores were out of target at first visit and; (c) subjects whose scores indicated they were bradykinetic at first visit. Comparisons between final scores in the two arms, as well as differences between first and final score were both planned. Assessments of number of visits was performed using a *χ*^2^ test. Mann–Whitney test was used when numbers were less than 35 and the distribution was not normal (Fig. 2).

### Reporting summary

Further information on research design is available in the Nature Research Reporting Summary linked to this article.

## Supplementary information

Supplementary Table 1

Reporting Summary

## Data Availability

Data are held in Dryad Digital Repository and the Digital Object Identifier will be made available on request to the corresponding author.
