# Supplementary material for: A blinded, controlled trial of objective measurement in Parkinson’s disease
Source: NPJ Parkinsons Dis. 2020 Nov 20;6:35. doi: 10.1038/s41531-020-00136-9 (PMC7680151; doi:10.1038/s41531-020-00136-9)
Supplement: Supplementary file 1 — Supplementary Table 1 [file 41531_2020_136_MOESM1_ESM.pdf]

Supplementary Table 1

Demographics of participants in each arm who completed study (Analysed) and who were Enrolled

	PKG-				PKG+			
	Analysed N=79		Enrolled N=103		Analysed N=75		Enrolled N=97	
	Mean±StD	95% CI.	Mean±StD	95% CI.	Mean±StD	95% CI.	Mean±StD	95% CI.
Age	67.4±4.9	66.2 - 68.3	67.4±4.7	66.4 - 68.3	68.2±4.5	66.8 - 69.1	68.0±4.7	67.1 - 69.0
Gender (F/M)	32/47		44/59		40/35		46/51	
Years to diag.	6.3±3.6	5.1 -6.8	6.5±4.0	5.8 - 7.3	6.4±4	5.1 - 6.9	6.4±4.2	5.5 - 7.2
LEDD	760±325	683 -8330	816±382	739 - 892	675±330	599 - 751	713±377	637 - 790
UPDRS I	11.2±6.1	9.8 -12.6	11.5±6.1	10.3 - 12.7	10.5±4.9	9.4 - 11.7	10.4±4.8	9.4 - 11.4
UPDRS II	10.7±6.5	9.2 -12.2	11.6±7.2	10.2 - 13.0	9.5±5.8	8.2 - 10.9	9.9±6.0	8.7 - 11.1
UPDRS III	35.8±11.3	33.4 -38.5	36.3±11.4	34.0 - 38.6	35.1±9.6	32.8 - 37.3	35.9±10.0	33.8 - 37.9
UPDRS IV	4.6±3.6	3.8 -5.4	4.8±3.7	4.1 - 5.6	5.0±3.8	4.1 5.9	5.3±4.0	4.5 - 6.1
UPDRS Total	62.3±19.6	57.9 -66.9	64.1±20.6	60.0 - 68.3	59.6±16.9	55.7 - 63.5	60.9±17.3	57.4 - 64.5
PDQ 39	29.6±19.1	25.2 -34.1	31.6±20.1	27.5 - 35.7	26.9±16.8	23.0 - 30.8	27.1±16.4	23.7 - 30.5
H&Y	2.0±0.6	1.9 -2.1	2.0±0.7	1.9 - 2.2	1.9±0.6	1.8 - 2.0	2.0±0.6	1.8 - 2.1
MoCA	25.9±2.5	25.3 -26.5	25.3±5.0	24.4 - 26.3	26.5±2.1	26.1 - 27.0	26.2±3.4	25.5 - 26.9
SENS PD	12.1±5.2	10.9 -13.3	12.3±5.4	11.2 - 13.4	11.1±4.9	10.0 - 12.3	11.1±4.7	10.2 - 12.1
NMS	9.7±4.9	8.6 -10.8	9.8±5.5	8.7 - 10.9	8.8±5.2	7.5 - 10.0	8.9±5.0	7.8 - 9.9
