# Supplementary material for: A blinded, controlled trial of objective measurement in Parkinson’s disease
Source: NPJ Parkinsons Dis. 2020 Nov 20;6:35. doi: 10.1038/s41531-020-00136-9 (PMC7680151; doi:10.1038/s41531-020-00136-9)
Supplement: Supplementary file 2 — Reporting Summary [file 41531_2020_136_MOESM2_ESM.pdf]

nature research

Corresponding author(s): Malcolm Horne

Last updated by author(s): Sep 24, 2020

Reporting Summary

Nature Research wishes to improve the reproducibility of the work that we publish. This form provides structure for consistency and transparency in reporting. For further information on Nature Research policies, see our Editorial Policies and the Editorial Policy Checklist.

Statistics

For all statistical analyses, confirm that the following items are present in the figure legend, table legend, main text, or Methods section.

n/a	Confirmed	
☐	☒	The exact sample size (*n*) for each experimental group/condition, given as a discrete number and unit of measurement
☐	☒	A statement on whether measurements were taken from distinct samples or whether the same sample was measured repeatedly
☐	☒	The statistical test(s) used AND whether they are one- or two-sided<br>*Only common tests should be described solely by name; describe more complex techniques in the Methods section.*
☒	☐	A description of all covariates tested
☒	☐	A description of any assumptions or corrections, such as tests of normality and adjustment for multiple comparisons
☐	☒	A full description of the statistical parameters including central tendency (e.g. means) or other basic estimates (e.g. regression coefficient) AND variation (e.g. standard deviation) or associated estimates of uncertainty (e.g. confidence intervals)
☐	☒	For null hypothesis testing, the test statistic (e.g. *F*, *t*, *r*) with confidence intervals, effect sizes, degrees of freedom and *P* value noted<br>*Give P values as exact values whenever suitable.*
☒	☐	For Bayesian analysis, information on the choice of priors and Markov chain Monte Carlo settings
☒	☐	For hierarchical and complex designs, identification of the appropriate level for tests and full reporting of outcomes
☒	☐	Estimates of effect sizes (e.g. Cohen's *d*, Pearson's *r*), indicating how they were calculated

Our web collection on statistics for biologists contains articles on many of the points above.

Software and code

Policy information about availability of computer code

Data collection	No soft ware was used to collect data but data was entered from paper onto Excel
Data analysis	Data analyses was performed using in house code in Excel or Stada/SE 16.0 for windows and Prism v 8 for graphs.

For manuscripts utilizing custom algorithms or software that are central to the research but not yet described in published literature, software must be made available to editors and reviewers. We strongly encourage code deposition in a community repository (e.g. GitHub). See the Nature Research guidelines for submitting code & software for further information.

Data

Policy information about availability of data

All manuscripts must include a data availability statement. This statement should provide the following information, where applicable:

- Accession codes, unique identifiers, or web links for publicly available datasets
- A list of figures that have associated raw data
- A description of any restrictions on data availability

Data is held in Dryad Digital Repository and the Digital Object Identifier will be made available on request to the corresponding author.

Field-specific reporting

Please select the one below that is the best fit for your research. If you are not sure, read the appropriate sections before making your selection.

☒ Life sciences ☐ Behavioural & social sciences ☐ Ecological, evolutionary & environmental sciences

For a reference copy of the document with all sections, see nature.com/documents/nr-reporting-summary-flat.pdf

Life sciences study design

All studies must disclose on these points even when the disclosure is negative.

Sample size	Power calculation based on Standard deviation Confidence Interval and effect size from pilot studies
Data exclusions	Data from subjects who failed to complete the study were excluded because a final measurement was required to measure the effect of the intervention.
Replication	Not relevent
Randomization	Randomisation not possible because of study design which was intended to overcome bias from doctors whose intervention was likely to be a greater source of bias. Attention was givent to the demographics to avoid bias from not randomising
Blinding	Investigators were blinded, Patients were blinded

Reporting for specific materials, systems and methods

We require information from authors about some types of materials, experimental systems and methods used in many studies. Here, indicate whether each material, system or method listed is relevant to your study. If you are not sure if a list item applies to your research, read the appropriate section before selecting a response.

Materials & experimental systems

n/a	Involved in the study	
☒	☐	Antibodies
☒	☐	Eukaryotic cell lines
☒	☐	Palaeontology and archaeology
☒	☐	Animals and other organisms
☐	☒	Human research participants
☐	☒	Clinical data
☒	☐	Dual use research of concern

Methods

n/a	Involved in the study	
☒	☐	ChIP-seq
☒	☐	Flow cytometry
☒	☐	MRI-based neuroimaging

Human research participants

Policy information about studies involving human research participants

Population characteristics	Male and female people with Parkinson's disease aged between 59 and 75 years and with Montreal Cognitive Assessment Score >21. Disease duration of 4 or more years or on 4 or more dose /day of levodopa. No contraindication to increasing the dose of levodopa and not currently receiving or under consideration for device assisted therapies.
Recruitment	Recruitment was by advertisement through advocacy associations and social media and the Florey institute's web site. There is a risk of bias through self selection but this would be small and unlikely to influence the results of the study
Ethics oversight	St Vincent's Hospital, Melbourne, Human Research & Ethics Committee (approval No. HREC/17/SVHM/298)

Note that full information on the approval of the study protocol must also be provided in the manuscript.

Clinical data

Policy information about clinical studies

All manuscripts should comply with the ICMJE guidelines for publication of clinical research and a completed CONSORT checklist must be included with all submissions.

Clinical trial registration	This Trial was registered on ANZCTR (https://www.anzctr.org.au/Trial/Registration/TrialReview.aspx?id=373960), registration number ACTRN12618000197235.
Study protocol	study protocol is available on request to the senior author

Data collection	Dat was collected in movement disorder clinics in Australia between March 2018 and December 2019
Outcomes	Primary and Secondary outcomes were conventional clinical scales that are recognised measures of imporved in motor scores and quality of life in Parkinson's disease
